# Neurointervention—from entry to expertise: Examining gender bias across different training access routes in Europe

**DOI:** 10.1177/15910199251336928

**Published:** 2025-04-29

**Authors:** Helena Guerreiro, Anne-Christine Januel, Franziska Dorn, Riitta Rautio, Anna A. Kyselyova, Razvan Alexandru Radu, João Reis, Jens Fiehler, Isabel Fragata

**Affiliations:** 1 Department of Diagnostic and Interventional Neuroradiology, 37734 University Medical Center Hamburg-Eppendorf, Hamburg, Germany; 2 Department Neuroradiology, 36760 University Hospital Toulouse, Toulouse, France; 3 Department of Neuroradiology, University Hospital Bonn, Bonn, Germany; 4 Department of Interventional Radiology, 60652 Turku University Hospital, Turku, Finland; 5 Department of Clinical Neurosciences, 87267 “Carol Davila” University of Medicine and Pharmacy, Bucharest, Romania; 6 Departments of Neurology and Interventional Radiology, University Emergency Hospital Bucharest, Bucharest, Romania; 7 Department of Neuroradiology, 90463 Centro Hospitalar Universitário de Lisboa Central, Lisboa, Portugal; 8 NOVA Medical School, Universidade Nova de Lisboa, Lisboa, Portugal

**Keywords:** Neurointervention, interventional neuroradiology, women in neurointervention, equality, leadership

## Abstract

**Background/purpose:**

Gender bias in academic medicine has been widely described. In Europe, training and career pathways in neurointervention (NI) are heterogeneous. We hypothesize that the access route to neuroradiology specialty and NI subspecialty may correlate with the proportion of women in the field and with their career progression.

**Methods:**

An online survey consisting of 18 questions was distributed through European professional societies and several online social platforms. A total of 422 responses from 54 different countries were collected and statistically evaluated.

**Results:**

Access routes to specialty and subspecialty did not correlate with the number of women practicing NI. However, men were significantly more likely to have children, to occupy leading positions, to have more clinical experience and higher weekly workload both in diagnostic and interventional neuroradiology. Female gender significantly affected career progression.

**Conclusion:**

This study reflects a positive change in European reality concerning gender bias. Distinct training access routes do not seem to affect the proportion of female neurointerventionalists. However, gender differences still negatively impact women NI careers, leading to lower workload, having less children, and a limited access to leading positions in NI.

## Introduction

The number of female medical students has outgrown the number of their male counterparts since the early 90s^1^ with recent overall percentages of 61 and 55 for Germany and Sweden respectively.^2^ Nevertheless, gender inequality in medicine remains a hot topic with several published articles from various clinical fields exposing gender disparities. An analysis across 35 years of graduating classes has shown that differences in promotion of female physicians persisted across every academic department.^3^ In fact, the proportion of female full professors in Germany and Sweden declined dramatically to 19% and 29% respectively.^2^ The exact number of females working in neurointervention (NI) remains unclear, partly due to the diverse training pathways leading to the subspecialty, and to the lack of an official supervising entity. Women represent only 10% of interventional radiologists^4^ and gender disparities in the field have not changed within the last decade.^5^ In Europe the overall proportion of female neurosurgeons—12%—is comparable.^6^ In 2019, the proportion of female members of the World Federation of Interventional and Therapeutic Neuroradiology was only 6%.^7^ To further illustrate the extent of gender disparity in NI, it is worth mentioning that the European Society of Minimally Invasive Neurological Therapy (ESMINT) annual congress had only one female president in its 15-year history. Despite recent efforts toward gender equity through the newly formed Women in NI committee (WIN@ESMINT), only two women have been part of its executive committee.

NI training pathways within Europe are extremely heterogeneous.^8^ In some countries, subspecialty access is reserved to radiologists, while some include neurologists and/or neurosurgeons. Portugal is the only country in Europe with an exclusive neuroradiology residency program, and only recently, a dedicated subspecialty program. However, many countries do not have a formal NI fellowship training program.^9^ Access to residency programs in Europe is equally heterogeneous, ranging from national blinded access exams to face to face selection interviews.

The aim of this study was to determine whether the access route to neuroradiology specialty and neurointerventional subspecialty training correlates with the proportion of women in the field of interventional neuroradiology and furthermore, whether this influences their career progression.

## Material and methods

### Data collection and statistical analysis

Participants responded to an online questionnaire composed of 18 questions (Supplemental Material S1). Responses were collected with the online platform Mentimeter (Stockholm, Sweden) from April to June 2023. This study was based on an anonymous online survey, and as such, it did not involve any procedures requiring Institutional Review Board approval. The questionnaire aimed to characterize the participants’ personal and professional background, access route to specialty and subspecialty training, weekly workload, and gender-related differences. Most questions were multiple choice with exception of questions 3, 4, 17 (open-ended questions), and questions 6 and 12 (rating scale input). Survey divulgation was done using social media (Twitter, LinkedIn, WhatsApp) and per e-mail, using the member database of the ESMINT and European Society of Neuroradiology.

Descriptive statistics and basic data visualization was performed with Microsoft Excel for Mac (Redmond, WA, USA). Frequencies and proportions are presented. Statistical analyses were performed with SPSS 27.0 (IBM, Chicago, IL, USA). Age groups were dichotomized into two groups to examine potential age-related differences: a younger group consisting of participants aged under 45 years and an older group comprising participants aged 45 years and older. Nonparametric variables were analyzed using chi-square independence test, Fisher's exact test for limited samples, Mann–Whitney U test for two independent variables. A *p*-value <.05 was considered significant.

## Results

The survey led to a total of 422 responses from 54 different countries over the course of two months and 22 days (83 days). Responses with no input on country or city were excluded (*N* = 37, 8.8%). Participants practicing outside geographical Europe (*N* = 37, 8.8%) or that did not answer the questions related to access to subspecialty training were excluded (*N* = 26, 6.2%).

### Demographics

A total of 152 women (47.2%) and 169 men (52.5%) were included in the analysis. One participant identified as nonbinary (0.3%). Participants from 33 different European countries answered the questionnaire. However, most answers came from Germany (*N* = 99, 30.7%), Portugal (*N* = 38, 11.8%), Italy (*N* = 26, 8.1%) and the United Kingdom (*N* = 22, 6.8%). Most participants were within the 30–54 age range (*N* = 266, 82.6%). Age group 25–39 had 10 participants (3.1%), 55–59 had 22 participants (6.8%) and 60–64 had 16 participants (5%). Only eight participants were older than 65 years (2.5%). Access to specialty was selected as interview (*N* = 137, 42.5%), blinded access exam (*N* = 73, 22.7%), curriculum vitae only (*N* = 36, 11.2%) or other nonspecified (*N* = 73, 22.7%). There were geographical differences in access to NI training for female and male NI, with apparently larger number of blinded access exams for female NI in our sample. Professional backgrounds were classified as following: Neuroradiology (*N* = 248, 77.3%), Radiology (*N* = 56, 17.4%), Neurosurgery (*N* = 12, 3.7%), Neurology (*N* = 5, 1.6%), or other (*N* = 1, 0.3%). Female NI in our survey were predominantly neuroradiologists.

Access to subspecialty training was done per fellowship application in 87 cases (27%) and nomination in 79 (24.5%). One hundred thirty-one participants answered *other* (40.7%) and 25 did not specify (7.8%). Job titles were described as attending (*N* = 45, 14%), consultant (*N* = 142, 44.1%), fellow (*N* = 32, 9.9%), head of department (*N* = 48, 14.9%), resident (*N* = 26, 8.1%), *other* (*N* = 8, 2.5%) or not specified (*N* = 1, 0.3%). One hundred thirty participants had a PhD academic title (41%), 106 had a masters degree (33.2%), 39 had a habilitation (12%), and 37 a professorship (11.6%); seven participants did not specify (2.2%). Notably, less women in eastern Europe countries had PhD titles, when compared to men in the same countries.

### Career path

**Figure 1.**
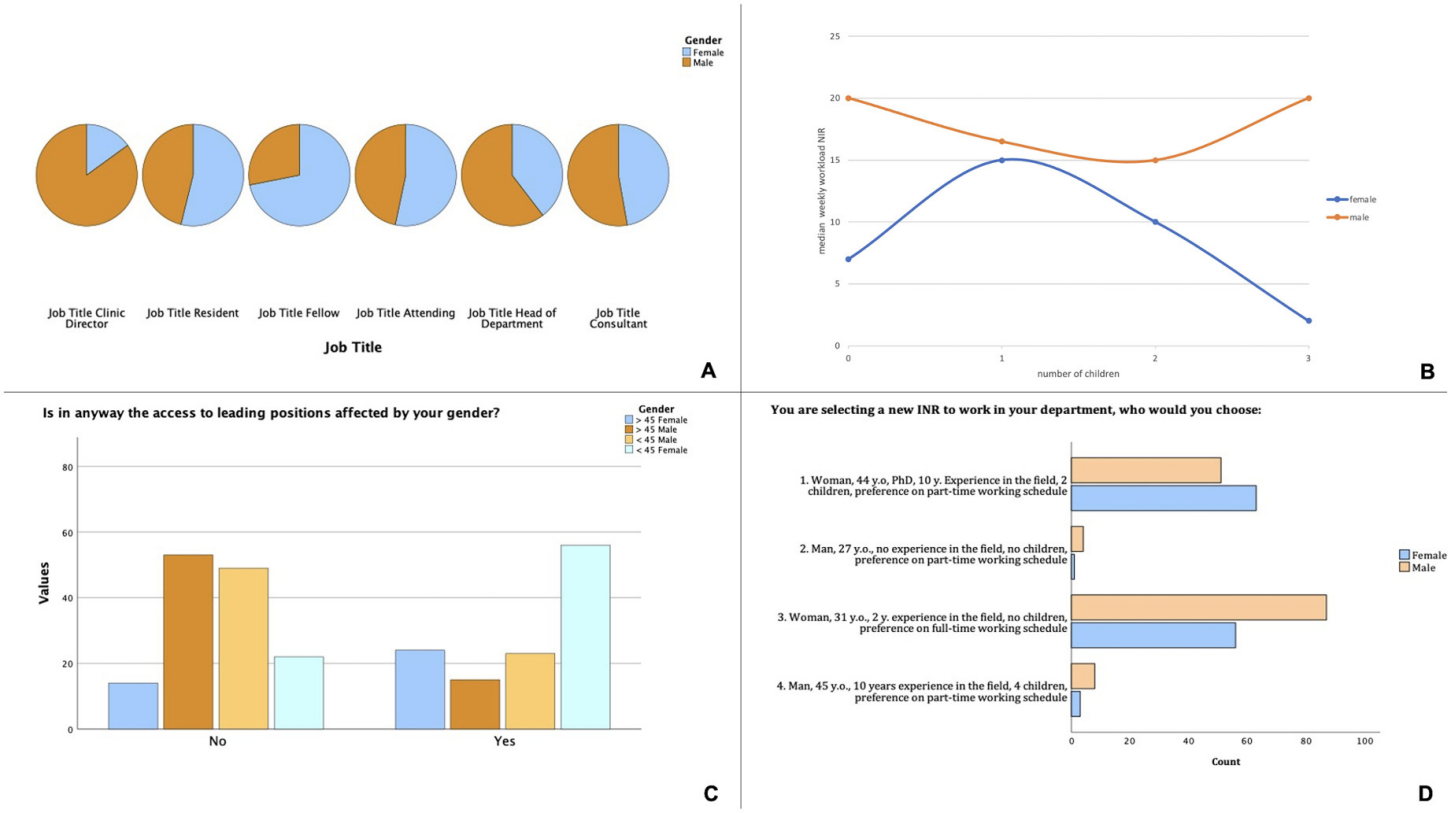
(A) Distribution of participants’ job titles according to their gender. Note the clear predominance of male head of departments and clinic directors. Most participating fellows were, however, female. (B) Distribution of the median weekly workload for female and male NI, according to the number of children. There is a globally lower week workload for female NI in our sample when compared to their male counterparts, a difference that increases as women have more children. (C) The majority of female NIR reported that their gender affected their career progression (69.7%), and this was true mostly for the participants under 45 years of age. Only 37% of male participants felt their gender affected their career progression. (D) Scenario-based question where participants were asked to choose from four different fictional candidates. There was no significant difference between female and male participants, with the majority choosing candidates 1 or 3. A preference toward full-time working schedule and/or academic achievements can be assumed.

Abbreviation: NI: neurointervention.

**Figure 2.**
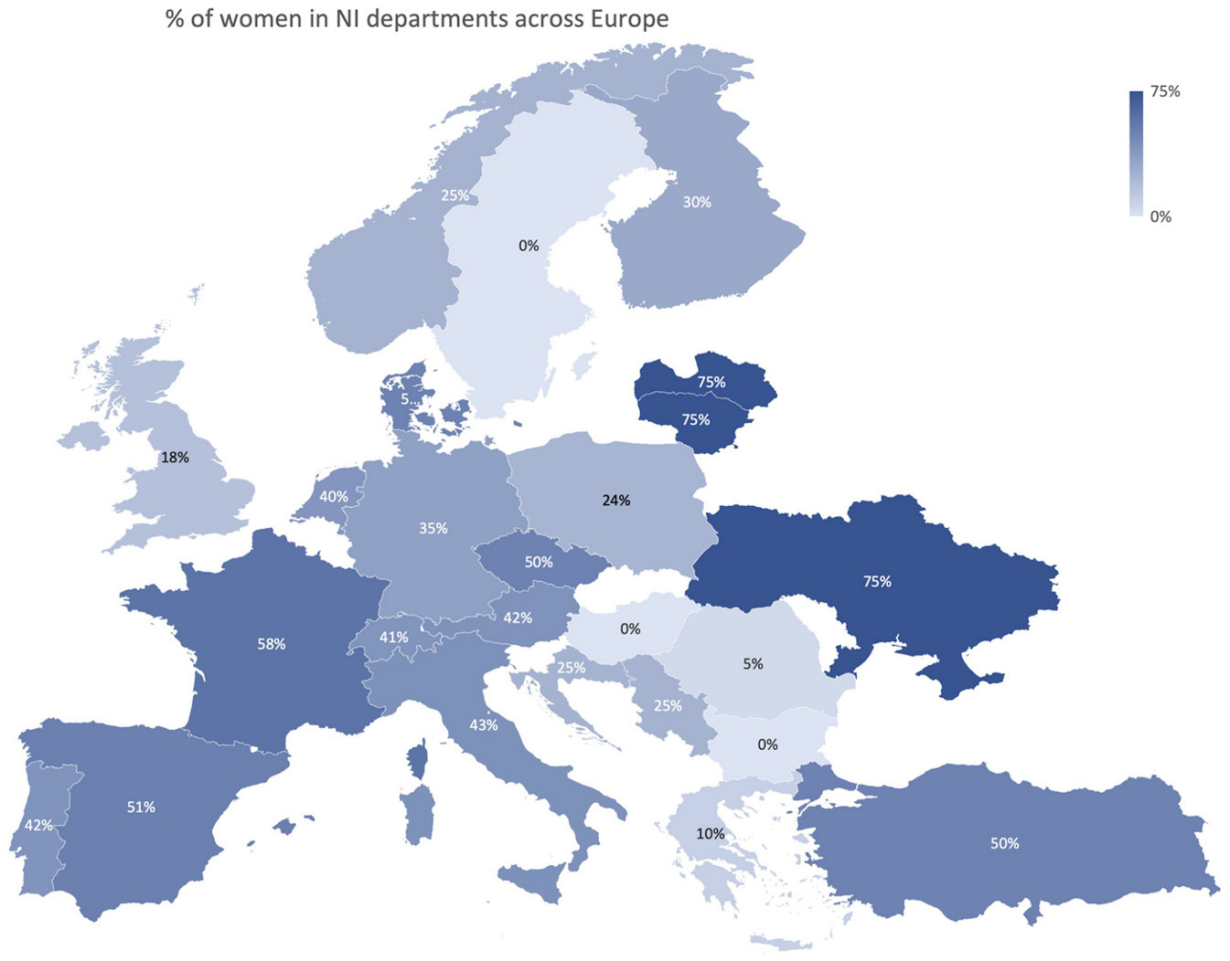
Geographical distribution of female NI in departments across Europe.

Abbreviation: NI: neurointervention.

Men were significantly more experienced than women (years practicing NI, mean 11.7 vs 8.9, *p* < .001). A significant association between job title and gender was observed (*p* < .001, Figure 1A). The number of female fellows was significantly higher (*n* = 23 vs 9, *p* = .013) whereas the number of male clinical directors was significantly superior (*n* = 17 vs 3, *p* = .011). There was no significant association between professional background, academic degree, and gender. The percentage of female NI in the departments had regional differences across Europe, with lower numbers of women in eastern European countries (Figure 2).

### Specialty and subspecialty training access routes

Different access routes to training did not significantly correlate with the proportion of women working in NI. There was no statistically significant evidence suggesting an association between academic title and training access route.

### Workload

**Figure 3.**
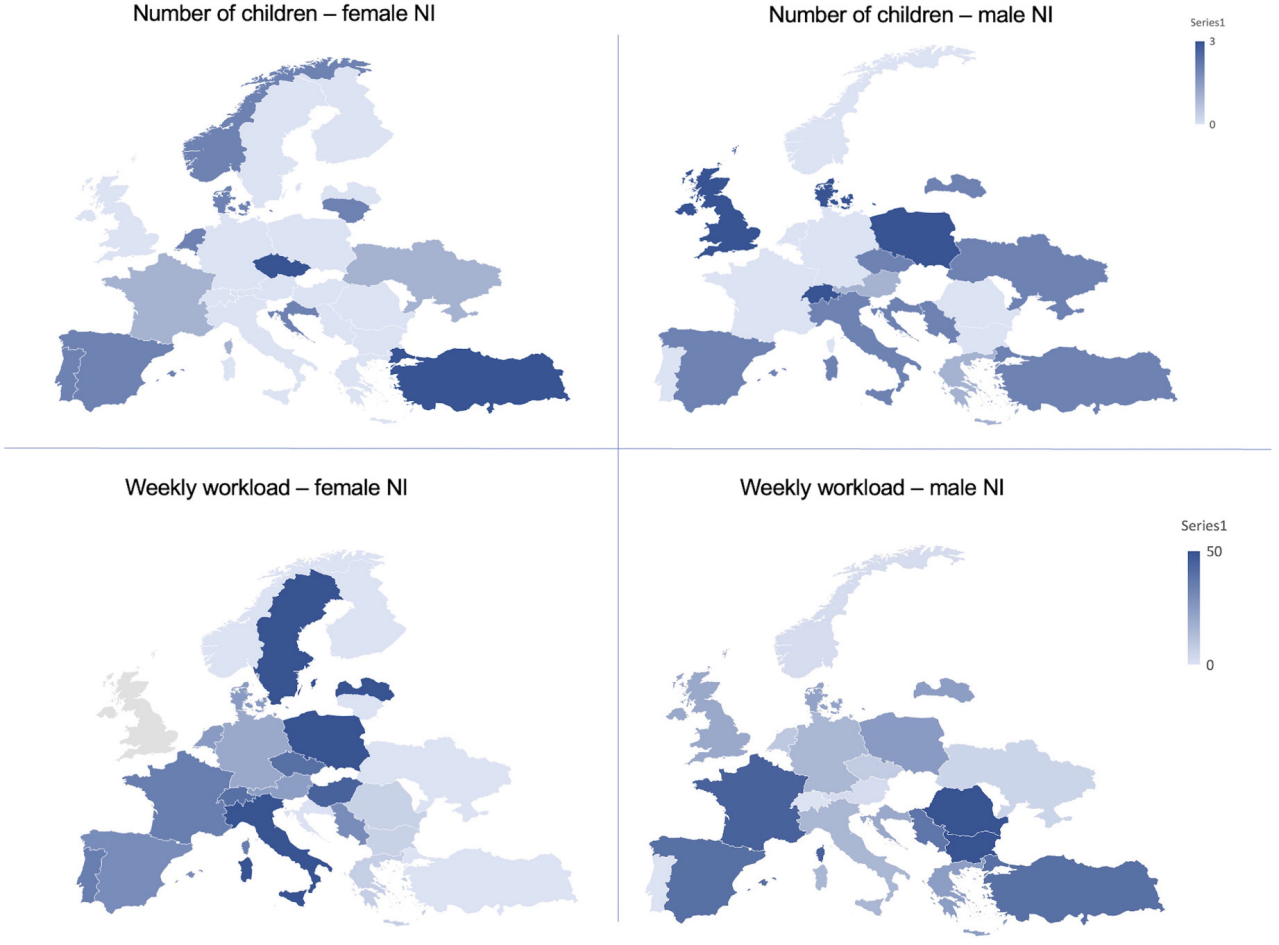
Geographical distribution of the number of children in male NI versus female NI (above) and of weekly workload (in hours per week) between male and female NI (below). Women had less children overall. Geographical differences were also found, showing that coincidentally in countries where men NI had more children, women NI had less children. Women NI have a significantly lower weekly workload in neurointervention, when compared to men across most European countries.

Abbreviation: NI: neurointervention.

Men had a significantly higher weekly workload in NI across all European countries (mean 19.6 vs 15.4, *p* = .005), but more strikingly in eastern European countries (Figure 3), whereas women dedicated significantly more time to diagnostic neuroradiology compared to men (mean 27.5 vs 21.8, *p* = .002). This difference was striking when correlated with the number of children (Figure 1B). Weekly workload in diagnostic and interventional neuroradiology did not significantly differ among younger and senior participants.

### Number of children

Number of children reported by participants ranged from 0 to 2. 105 participants had no children (32.6%), this was the case especially for women (*n* = 67, 63.8%), when compared to male participants (*n* = 38, 36.2%). Men had significantly more children than women (mean 1.2 vs 0.68 respectively, *p* < .001). Geographical differences were found, showing that coincidentally in countries where male NI had more children, female NI had less children (Figure 3). The number of children did not significantly differ among academic backgrounds or specialty access routes. Job titles correlated significantly with the number of children: heads of department, attendings, fellows, and residents had a higher likelihood of having no children while consultants were more likely to have one child or more (*p* < .001).

### Gender bias

Only 29 participants were asked about family planning during job interviews with no significant difference among genders in absolute numbers (*N* = 18 for women, *N* = 11 for men, *p* = .97). However, a geographical difference was evident, where female NI were asked about family planning mostly in Germany, Ukraine, and Bulgaria. Younger female participants (< 45 years old) were more frequently asked about family planning than their older counterparts (*N* = 17 vs *N* = 1, *p* = .003). Access to leading positions correlated significantly with gender (*p* < .001) with most men reporting their gender did not affect their career progression (*N* = 1055, 62.7%) whereas 80 women reported that it did affect their career progression (69.7%), and this was true especially for younger participants (*N* = 56 vs *N* = 25, *p* = .003) (Figure 1C). Most heads of department were male (*N* = 228, 70.8% vs *N* = 59, 18.3% female). In the question where the participants were asked to choose from four different candidates, no significant differences were observed between men and women or among age groups (Figure 1D). A total of 143 participants chose option 3 (52.2%) and 115 chose option 1 (42%).

## Discussion

In the last decades, we have witnessed a shift in gender distribution in Medicine, with increasing numbers of female medical students and female physicians. However, some specialties are still traditionally dominated by male physicians, such as surgical specialties, mostly because they are physically demanding and difficult to combine with a family life. Interventional neuroradiology shares some of the challenges of surgical specialties for women, with the added risk of radiation exposure in reproductive years.^7^

Whether the underrepresentation of women in interventional neuroradiology is a result from the inherent difficulties of practice, or from training selection processes, is unknown.

Neurointerventional training in Europe is highly heterogeneous. In many countries, there is still no formal training program, and in countries with such programs, different access conditions exist.^8^ In a recent survey from 2020 by Pizzini et al.^8^ although most countries demand specific NI requirements, only Germany, Greece, Hungary, Iceland, the Netherlands, Poland, Romania, Switzerland, and Turkey have a specific NI qualification. However, the way to access qualification programs is not specified. This was one of the goals of this survey: to evaluate how access routes to NI training may affect the access of women to this field. Access to postgraduate training programs commonly involves a personal interview, that not only evaluates the curriculum of the candidate, but also his/her personal profile, seeking for a perfect match, while inadvertently opening the possibility for a selection bias. A majority of men, slightly more than 50%, answered the survey, possibly reflecting the present interest and consciousness of gender bias problems in the NI community. However, the almost 50% of female representativeness surely does not reflect the real proportion of female NI, but rather a gender motivation to this topic.

The data reported here yield important information but are likely affected by various biases. Most participants were younger than 55, possibly reflecting the ease of access to social media, through which the survey was sent. However, more than two-thirds of participants were senior positions—most were consultants which may be associated with the fact that senior NIR are usually more active within the professional societies, which distributed this survey. The majority of participants (77%) were neuroradiologists, which reflects the predominant practice in Europe,^8^ and this was especially true for female NI. This contrasts with the current practice in the United States, where around 51% of INR program directors are neurosurgeons, and 33% are radiologists.^10^

Access to subspecialty training in INR is very heterogeneous in Europe. Approximately one-third of the participants reported access to a dedicated fellowship through an application process, but one-third was nominated, meaning there was no objective selection process. Most replies were “other,” implying there are even more different ways to enter the subspecialty across the Europe, and highlighting the need for a European convergence on fellowship programs and routes of access.

Access by interview was more common than by blinded access exam or CV only, and therefore we initially hypothesized that this could induce gender bias and negatively influence the access of women to NI training.

The interview process of selection aims to better assess the applicant and his/her profile, in order to choose the most suitable candidate. NI is a demanding job, comparable to any surgical specialty, and therefore, many interview questions might focus on personal life as well as motivation for taking the subspecialty. For women, these questions often involve family planning, since pregnancy and breast-feeding many times imply the temporary suspension of activity in the angiosuite. Unconscious biases have been identified during the interview process in fellowships.^11^ Examples are the affinity bias, where there is the tendency to prefer someone similar to the interviewer—which might be relevant considering the significant majority of NI program directors are men (over 90% in the United States),^10^ or the maternal bias, a negative assumption about a woman's performance or commitment based on her status as a parent or potential parent. Many examples have shown that “blind” interviews lead to increases in hiring of women—such as the “blind” auditions in symphony orchestras, that led to increased hiring of women musicians.^12^ With this being said, we found, however, no relation between different access routes to NI training and the proportion of women working in NI, in our European sample. Additionally, it is fortunate that only 29 out of 137 (21%) participants that had an interview as part of the selection process, were queried about family planning. These were of course the younger participants, but both men and women, without significant difference between genders. From our analysis, it seems that although access to subspecialty training is heterogeneous and bias-prone, this does not affect the access of women to training in NI in Europe. In fact, most fellows in our survey were females, probably reflecting increasing numbers of female trainees. Also, a higher number of female NI reported access by blinded exam, which is possibly explained by the younger age group and recently implemented training programs, with formal access exams. However, the number of fellows and gender distribution across Europe are unknown, or at least, are not centralized by any scientific society or organization.

In our survey, there was a significant association between job title and gender, and although half of participants were male, almost all directors were also male, with only three female NI in leading positions. This mirrors the United States, with 92% male program directors,^10^ and previous results on gender gap in NI, reporting less than 20% of female supervisors in NI in Europe.^7^

Male NI did not seem to feel any impact of their gender in their career progression, but that was the reverse for women, especially for female NI under 45 years. This is probably reflected in the number of children, since in this relatively gender balanced sample, men had significantly more children than women. In fact, of the participants with no children, women were the majority. These results are in line with previous studies^7^ and are transversal to many other surgical specialties.^13,14^ Interestingly, in our survey, countries where men have more children correspond to countries where female NI had less children, which probably reflects cultural differences across European countries. Despite having more children, men were still able to carry heavier workloads in NI, underlining the assumption that their gender did not seem to affect their career progression.

In response to the question where the participants were asked to choose from four possible job candidates, most chose the only candidate willing to work full-time. This reflects the deep sitting practice of choosing medical graduates willing to sacrifice their personal lives to a fully committed work-life. Flexible working arrangements in their different forms are believed to be crucial for an inclusive working environment, enabling career advancements and promotion while effectively managing family responsibilities for both men and women.^15^ Academic achievements and clinical experience of the second most voted candidate seemed to also play a role. Characteristics such as full-time availability and academic endeavors seem to be of higher importance than the gender. However, it's important to acknowledge that these can inadvertently hinder female participation and representation in our field.

So, although female physicians seem to have equal access to training, difficulties arise during their career pathway, reducing their capability for weekly workload in NI, but also impacting on their personal lives, particularly on the number of children, or having children at all, and limiting their access to leading roles in NI. This again reminds us of the importance to fight the “leaky pipeline” phenomenon,^16^ whereby women leave their careers and fail to reach top leadership positions, when compared to men.

Despite the large number of participations, our survey did not sample all countries equally, and this limits the interpretation of the geographical results. Also, we are unaware of each country's formal access to NI training. As discussed, there is an overrepresentation of female NI participants, which most likely influenced results, especially concerning subjective questions. The nature of this study may be subject to response bias. Question wording and its interpretation by the participants may be an additional source of bias. Finally, the inclusion of countries outside Europe, namely Middle Eastern countries, and Eastern countries would be of interest, possibly revealing even greater disparities of access to training and career progression between female and male NI.

In this European survey, we found that female physicians seem to have equal access to training in NI, but less weekly workload, less children, and limited access to leading roles in NI. The exact number and gender distribution of NI fellows in Europe are unknown.

## Supplemental Material

sj-docx-1-ine-10.1177_15910199251336928 - Supplemental material for Neurointervention—from entry to expertise: Examining gender bias across different training access routes in Europe Supplemental material, sj-docx-1-ine-10.1177_15910199251336928 for Neurointervention—from entry to expertise: Examining gender bias across different training access routes in Europe by Helena Guerreiro, Anne-Christine Januel, Franziska Dorn, Riitta Rautio, Anna A. Kyselyova, Razvan Alexandru Radu, João Reis, Jens Fiehler and Isabel Fragata in Interventional Neuroradiology
