# Supplementary material for: Neurointervention—from entry to expertise: Examining gender bias across different training access routes in Europe
Source: Interv Neuroradiol. 2025 Apr 29:15910199251336928. Online ahead of print. doi: 10.1177/15910199251336928 (PMC12040865; doi:10.1177/15910199251336928)
Supplement: Supplementary file 1 [file sj-docx-1-ine-10.1177_15910199251336928.docx]

Question	Possible Answers
1. What is your gender?	Female
	Male
	Other
2. What is your age group?	25-29
	30-34
	35-39
	40-44
	45-49
	50-54
	55-59
	60-64
	> 65
3. In which country do you practise?	open answer
4. In which city do you live?	open answer
5. What is you professional background?	Radiology
	Neuroradiology
	Neurology
	Neurosurgery
	Other
6. How long have you been practising NIR?	scale
7. What is your professional title?	Resident
	Fellow
	Attending
	Consultant
	Head of Department
	Clinic Director
	Other
8. What is your academic degree?	Masters
	PhD/Promotion
	Habilitation
	Professorship
9. How did you access speciality training?	Blinded access exam
	Interview
	CV only
	Other
10. If the access was done per interview, were there any questions regarding family planning?	Yes
	No
	I don't remember
11. How was the access to sub-speciality training in NIR?	Fellowship Application
	Nomination
	Other
12. What is your weekly workload (in hours):	Neurointervention (scale)
	Diagnostic Neuroradiology (scale)
13. How many children do you have?	0
	1
	2
	3 or more
14. What is the approximate percentage of women working in your department?	0%
	10%
	25%
	50%
	75%
	100%
15. What is the gender of your head of department?	Male
	Female
	Other
16. Do you think the access to a leading position is in anyway affected by your gender?	Yes
	No
	I don't know
17. If yes, in which way?	open answer
18.You are selecting a new INR to work in your department, who would you choose:	Woman, 44 y.o, PhD, 10 y. Experience in the field, 2 children, preference on part-time working schedule
	Man, 27 y.o., no experience in the field, no children, preference on part-time working schedule
	Woman, 31 y.o., 2 y. experience in the field, no children, preference on full-time working schedule
	Man, 45 y.o., 10 years experience in the field, 4 children, preference on part-time working schedule
